# Long-Term Effects of COVID-19 on Optic Disc and Retinal Microvasculature Assessed by Optical Coherence Tomography Angiography

**DOI:** 10.3390/diagnostics15010114

**Published:** 2025-01-06

**Authors:** Mine Ozturk, Deniz Kumova Guler, Ekin Ece Oskan, Feyza Onder

**Affiliations:** 1 Department of Ophthalmology, Haseki Training and Research Hospital, 34096 Istanbul, Turkey; denizkumova82@gmail.com (D.K.G.); onderfeyza@yahoo.com (F.O.); 2 Department of Ophthalmology, Kirikhan State Hospital, 31440 Hatay, Turkey; ekineceoskan@gmail.com

**Keywords:** long-term, SARS-CoV-2, COVID-19, OCTA, retina

## Abstract

**Objectives**: To evaluate the long-term effects of coronavirus disease (COVID-19) on optic disc and macular microvasculature. **Methods**: 40 post-COVID-19 and 40 healthy subjects were included. Optical coherence tomography angiography (OCTA) was performed for all subjects at the first visit and repeated in the fourth and twelfth months. Radial peripapillary capillary (RPC) vessel density (VD), retinal nerve fiber layer (RNFL) thickness, foveal avascular zone (FAZ) area, FAZ perimeter, VDs of the fovea, parafovea, and perifovea at superficial capillary plexus (SCP) and deep capillary plexus (DCP), and central macular thickness (CMT) were evaluated. The OCTA measurements of the COVID-19 group were compared with the control group. **Results**: The COVID-19 group showed lower VD values than the control group in the nasal parafoveal quadrant of the SCP at all visits (*p* = 0.009, *p* = 0.47, *p* = 0.042) and in the superior perifoveal quadrant of the DCP in the twelfth-month visit (*p* = 0.014). At all visits, FAZ area and FAZ perimeter were higher (*p* = 0.02, *p* = 0.02, *p* = 0.002; *p* = 0.002, *p* = 0.003, *p* = 0.005), foveal VD values of both SCP and DCP were lower (*p* < 0.001, *p* < 0.001, *p* < 0.001; *p* = 0.005, *p* = 0.001, *p* = 0.001), and CMT was lower (*p* < 0.001, *p* = 0.001, *p* = 0.001) in the COVID-19 group. The COVID-19 group had higher temporal quadrant RPC at all visits (*p* = 0.003, *p* = 0.003, *p* < 0.001) and higher average, superior and inferior RNFL at first and fourth-month visits (*p* = 0.014, *p* = 0.020; *p* = 0.001, *p* = 0.003; *p* = 0.021, *p* = 0.024). **Conclusions**: There are long-term changes that mainly point to the ischemia in the COVID-19 patients. We emphasize the need for long-term ophthalmologic and systemic follow-up of COVID-19 patients regarding potential complications.

## 1. Introduction

The novel coronavirus disease 2019 (COVID-19) was first reported in Wuhan, China, in December 2019 [1]. Since 11 March 2020, when it was declared a pandemic by the World Health Organization, millions of people have been infected with the SARS-CoV-2 virus. After the end of the pandemic period, which led to millions of acute cases and thousands of deaths, an additional noteworthy phenomenon emerged, namely the presence or continuation of symptoms after the acute phase of the SARS-CoV-2 infection. Such continued symptoms are commonly referred to as long COVID [2] or post-COVID-19 [3], and more than 100 post-COVID-19 symptoms related to multiple systems (e.g., respiratory, neurologic, musculoskeletal, and cardiovascular) have been described [4]. Since the long-term complications of COVID-19 are still not fully clear, studies on this subject are continuing.

Microvascular changes in various organs, such as lungs, skin, and retina, in COVID-19 patients have been reported in previous studies [5,6]. SARS-CoV-2 has been associated with generalized coagulopathy and organ dysfunction [7]. It binds with high affinity to angiotensin-converting enzyme 2 (ACE2) receptors on all endothelial cells. Activating the renin/angiotensin system causes downregulation of ACE2 expression and an increase in angiotensin II, a strong vasoconstrictor, inhibitor of plasminogen activator, and enhancer of tissue factor expression, resulting in thrombosis. It also initiates complement activation by producing reactive oxygen species and downregulating the C1 inhibitor, which enhances cytokine release and vascular permeability and, hence, amplifies its thrombotic action [8,9].

The presence of the SARS-CoV-2 virus in the retina has been shown by real-time polymerase chain reaction (RT-PCR) in cadavers [10]. Previously, the SARS-CoV-2 entry ACE 2 was detected in the vitreous body and different retina cell types, including photoreceptor cells, Müller cells, and retinal vascular endothelial cells [11,12]. Retinal vascular pathologies secondary to COVID-19 have been reported in previous studies and some meta-analyses [13,14,15,16]. In addition to studies carried out in adult populations, similar investigations have also been conducted in pediatric COVID-19 groups [17]. Optical coherence tomography angiography (OCTA) is a noninvasive method for visualization of retinal microvasculature and may present a noninvasive and valid biomarker of microvascular dysfunction following COVID-19 disease [18]. The long-term effects of COVID-19 on retinal vasculature are still unknown. This study aims to evaluate the changes in the optic disc and retinal vasculature during a one-year follow-up and report the long-term results.

## 2. Materials and Methods

### 2.1. Study Design and Participants

This prospective cohort study was designed at the Haseki Training and Research Hospital, Istanbul, Turkey. Hospital medical records of Turkish patients hospitalized with a COVID-19 diagnosis confirmed with a positive RT-PCR analysis between 1 February and 1 May 2021 were reviewed. For all patients, a post-treatment negative PCR test was confirmed before discharge. Patients admitted to the intensive care unit, patients with a smoking history, and patients with systemic diseases, such as diabetes mellitus, coronary artery disease, hypertension, and obstructive or restrictive pulmonary disease, were excluded because of the reason that all three factors may affect the vascular system and the results of an OCTA scan. Novel coronavirus pneumonia (NCP) was classified as mild to moderate or severe. Mild to moderate NCP was characterized by two criteria: (1) clinical manifestations, including fever, joint or muscular discomfort, sore throat, and/or cough, along with an SpO_2_ level exceeding 90% in ambient air, and (2) mild to moderate abnormalities observed on a computed tomography (CT) scan. Severe NCP was characterized by two criteria: (1) symptoms include fever, joint or muscle discomfort, sore throat, and/or cough, with a SpO_2_ level of ≤90% in ambient air, and (2) CT results indicative of bilateral diffuse pneumonia. Fifty-seven patients without exclusion criteria were invited by telephone to join this study 4 weeks after discharge from the hospital with at least one negative PCR report, and 45 subjects agreed.

Another inclusion criterion was body mass index (BMI), which may affect OCTA results. Patients with a BMI between 15 and 25, the normal value, were included in the study, and one patient who did not meet the criteria was excluded. The remaining 44 patients underwent a complete eye examination in the first month after discharge from the hospital, including a dilated fundus examination with 1% tropicamide. The ophthalmologic exclusion criteria were high hyperopia and high myopia (greater than 6 diopters), congenital eye disease, macular diseases, retinal vascular diseases, optic nerve diseases, such as glaucoma or optic neuritis, previous ocular surgery other than uncomplicated cataract surgery, and significant lens opacity causing low-quality OCTA images. After an eye examination, two patients were excluded due to macular dystrophy and amblyopia. Then, 42 patients were evaluated using OCTA, and two were excluded due to poor-quality scans. Forty patients were included in the study and asked to come for visits during the fourth and twelfth months after discharge from the hospital. Complete ophthalmologic examination and OCTA scan were planned for each follow-up visit. The healthy control group, with the same exclusion criteria, consisted of 40 age- and sex-matched Turkish controls selected from willing medical personnel with a negative PCR test. A complete ophthalmic examination and OCTA scan were performed on the healthy control group at first, fourth, and twelfth-month visits. All participants’ right eyes were included.

The OCTA measurements of the COVID-19 group were compared with those of the healthy control group.

COVID-19 vaccinations and recurrent COVID-19 infection during the 1-year follow-up period were recorded.

### 2.2. OCTA Assessments

All OCTA scans were performed using the AngioVue Imaging System version 2017.1 (Optovue. Inc., Fremont, CA, USA) by the same experienced ophthalmologist (MO) at the same time of day (between 9 a.m. and 12 a.m.) following pupil dilatation with tropicamide. A 4.5 × 4.5 mm^2^ area centered on the optic disc was used for the optic disc scan, and a 6 × 6 mm^2^ area centered on the fovea was used for the macular area. Vessel density (VD) was defined as the ratio of the area occupied by vessels to the total area. Low-quality scans (signal strength index < 7) were excluded and repeated until satisfactory image quality (≥7) was obtained.

For the optic disc evaluation, radial peripapillary capillary (RPC) small vessel (SV) vessel density (VD) as the whole image VD (an area scan of 4.5 × 4.5 mm^2^), VD inside disc, and peripapillary VD (measured in a 750 μm wide annulus extending outward from the boundary of the optic disc) was calculated automatically by the software. The peripapillary region was divided into 4 segments: superior, inferior, nasal, and temporal. Average peripapillary retinal nerve fiber layer (RNFL) thickness and RNFL thicknesses in four quadrants (superior, inferior, nasal, and temporal) were also evaluated.

For the macula evaluation, foveal avascular zone (FAZ) parameters, including the FAZ area and perimeter circumference of the FAZ (PERIM), and the vessel density (VD) parameters, including the VD of the fovea, parafovea, and perifovea at the levels of both the superficial capillary plexus (SCP) and deep capillary plexus (DCP) were calculated automatically by the software. Angio-retinal scans were automatically positioned in three concentric rings with 1 mm, 3 mm, and 6 mm diameters centered on the fovea. The foveal region was the circular area with a diameter of 1 mm. The parafovea was the area between the inner (1 mm) and middle (3 mm) rings, while the perifovea was described as the area between the 3 mm and 6 mm radius. Parafoveal and perifoveal regions were further automatically divided into four segments: superior, nasal, inferior, and temporal. Microvascular flow parameters from the choriocapillaris (CC) and outer retina were also calculated. The software utilized previously characterized choroidal and retinal layers for automatic identification [17]. The upper and lower borders of the SCP were 3 µm beneath the internal limiting membrane and 15 µm beneath the inner plexiform layer, respectively. The DCP was delineated as the region between 15 µm and 70 µm underneath the inner plexiform layer. The upper and lower borders of the CC layer were established at 30 µm and 60 µm under the retinal pigment epithelium, respectively. Central macular thickness (CMT) was measured using the retina map mode.

### 2.3. Statistical Analysis

Statistical analysis utilized SPSS 15.0 for Windows applications. The descriptive statistics were given as mean, standard deviation, minimum, maximum, and median for numerical variables and percentages and numbers for categorical variables. When the normal distribution requirement was met, independent two-group comparisons of numerical variables were performed using the Student *t*-test; otherwise, the Mann–Whitney U test was used. Differences between dependent groups were evaluated using the paired *t*-test when the normal distribution requirement was met and the Wilcoxon Test when it was not. The alpha significance level was accepted as *p* ˂ 0.05. Multiple intragroup comparisons were performed with Bonferroni adjustment, and *p* was statistically significant if <0.016.

## 3. Results

Forty COVID-19 patients (40 eyes) and 40 healthy subjects (40 eyes) were included in the study. Of the 40 COVID-19 patients, 24 (60%) were male and 16 (40%) were female, and of the 40 healthy subjects, 24 (60%) were male and 16 (40%) were female. The mean age of the recovered COVID-19 patients was 42.9 ± 10.9 years (range: 18–58), and the mean age in the healthy control group was 40.1 ± 11.2 years (range: 21–58). The groups did not differ significantly in age (*p* = 0.053) or sex (*p* = 1.000). Table 1 shows the demographic data of the study participants.

All patients in the COVID-19 group had mild to moderate pneumonia on CT scan. At hospitalization, the mean peripheral capillary oxygen saturation (SpO_2_) was 93.2 ± 2.7%. Twenty-two patients (55%) had face-mask oxygen supplementation with a mean duration of 7.7 ± 1.8 days. None of the patients had been admitted to the intensive care unit (ICU) or required invasive ventilation because of respiratory failure during hospitalization.

All patients underwent a complete eye examination. None of the eyes had signs of ocular inflammation, and the examinations of the anterior and posterior segments were within the normal range.

**Figure 1:**
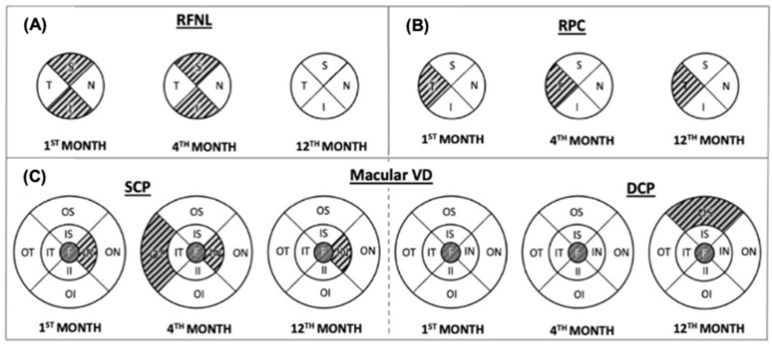
Retinal nerve fiber layer (RNFL) (**A**), radial peripapillary capillary (RPC) plexus (**B**), and macular vessel density (VD) in the superficial capillary plexus (SCP) and deep capillary plexus (DCP) (**C**) differences between COVID-19 and control group during one-year follow up. Lined areas show a significant difference. S: superior, N: nasal, I: inferior, T: temporal, F: fovea, IS: inner superior, IN: inner nasal, II: inner inferior, IT: inner temporal, OS: outer superior, ON: outer nasal, OI: outer inferior, OT: outer temporal.

The peripapillary parameters of both study groups are summarized in Table 2. The recovered COVID-19 patients had increased average, superior, and inferior quadrant RNFL values at first- and fourth-month visits compared with the healthy subjects (*p* = 0.014, *p* = 0.020; *p* = 0.001, *p* = 0.003; *p* = 0.021, *p* = 0.024, respectively). At the twelfth-month visit, all RNFL thicknesses had become similar to those of the control group (*p* = 0.213, *p* = 0.057, *p* = 0.074) (Figure 1A). Of the optic disc VD parameters, the temporal quadrant RPC was higher in all visits compared to the control group (*p* = 0.003, *p* = 0.003, and *p* < 0.001, respectively) (Figure 1B).

Table 3 compares the 2 groups’ FAZ, microvascular flow parameters, and CMT. The mean FAZ area and the FAZ PERIM values in the recovered COVID-19 patients were significantly higher than the mean FAZ area and the FAZ PERIM values in the healthy control group at all visits during 1-year follow-up (*p* = 0.002, *p* = 0.002, *p* = 0.002 and *p* = 0.002, *p* = 0.003, *p* = 0.005, respectively). Microvascular flow parameters were similar between the two groups at all visits. CMT in the recovered COVID-19 patients was significantly lower compared to the control group at all three visits (*p* < 0.001, *p* = 0.001, *p* = 0.001).

Table 4 compares the 2 groups’ SCP VD parameters. The recovered COVID-19 group had significantly lower foveal and parafoveal nasal VDs at all three visits (*p* < 0.001, *p* < 0.001, *p* < 0.001 and *p* = 0.009, *p* = 0.047, *p* = 0.042, respectively) compared to the control group. Perifoveal temporal SCP VD was lower in the fourth-month visit compared with the control group (*p* = 0.041) but similar in the twelfth-month visit (*p* = 0.342) (Figure 1C).

DCP VD parameters of both study groups are summarized in Table 5. Foveal VD in the recovered COVID-19 group was significantly lower compared with the control group at all three visits (*p* = 0.005, *p* = 0.001, *p* = 0.001, respectively). A progressive decrease was observed in the DCP VD in the perifoveal superior quadrant, which was not significant in the first and fourth months but reached significant values at the end of 1 year (*p* = 0.275, *p* = 0.384, *p* = 0.014 (respectively) (Figure 1C).

Between the fourth and twelfth-month visits, 39 patients received the CoronaVac vaccine. All healthcare workers in the control group were vaccinated with the CoronaVac vaccine. The average number of vaccinations was 2.7 for the COVID-19 group and 2.9 for the control group. During the 1-year follow-up, none of the participants had a second COVID-19 disease.

## 4. Discussion

Several studies have analyzed retinal microvasculature after COVID-19 disease with OCTA in different post-COVID periods.

Studies that analyzed patients at least 2 weeks post-hospital discharge found changes such as enlarged FAZ and reduced VD. Turker et al. analyzed 54 eyes of 27 recovered COVID-19 patients 1 week post-discharge and found lower VD in the superior and nasal quadrants of the SCP and all quadrants of the DCP, as well as higher choriocapillaris flow [19]. Gonzales-Zamora et al. studied COVID-19 patients 2 weeks after hospital discharge. They observed an enlarged FAZ area and lower foveal VD in both SCP and DCP compared to healthy subjects [6]. In the study by Abrishami et al. [20], OCTA was performed on patients after at least 2 symptom-free weeks post-COVID-19. The COVID group’s mean SCP VD and DCP VD were significantly lower than those of the control group in both the foveal and parafoveal regions. Although the FAZ area was larger in the COVID group, there was no statistically significant difference.

Another group of studies analyzed retinal vascular changes in patients up to 4 months post-COVID-19. In the Hazar et al. study, the VDs in the superior hemi, superior, and inferior quadrants were significantly lower (*p* = 0.033, *p* = 0.029, and *p* = 0.042) in SCP in the COVID-19 patients 1 month post-discharge. VDs were also significantly lower in the superior hemi quadrant, parafovea, and superior quadrant (*p* < 0.001, *p* = 0.026, and *p* = 0.004) in DCP. There was no difference between the COVID-19 and control groups in the mean FAZ value [21]. A study by Beni et al. involved 51 patients who had recovered from COVID-19, with a time frame of 40 to 95 days following their diagnosis. Compared to healthy subjects, patients with moderate-intensity SARS-CoV-2 pneumonia exhibited increased superficial and deep macular VDs in the perifoveal and parafoveal regions [22]. This study contradicts other studies that reported a reduction in VD in the eyes of post-COVID patients [18,19,23]. The study by Erogul et al. included 32 COVID-19 patients with ≥1 month of recovery and found significantly lower SCP VDs in all foveal regions than in the control group. Foveal DCP VD in the COVID-19 group was also significantly lower than in the control group. Compared to the control group, FAZ parameters were higher in the COVID-19 group. Significant differences existed in the foveal VD and FAZ area but not in the FAZ perimeter. Choriocapillaris and outer retina flows were significantly lower in the COVID-19 group [24]. On the contrary, some other studies did not find any retinal changes. Brantl et al. found no retinal changes in patients three months post-COVID recovery [25]. Also, Szkodny et al. found nothing significant on OCTA in the group of 156 COVID patients compared with 98 healthy subjects 1–4 months post mild to severe diagnosis of COVID-19 [26]. Zapata et al.’s study included 69 post-COVID-19 patients and divided them into three groups: mild (outpatient), moderate, and severe. They found that fovea-centered VD was reduced in moderate and severe groups compared with mild and control groups [27].

Other studies investigated OCTA findings in patients up to eight months post-COVID-19. Cennamo et al. found a significant reduction in the DCP, SCP, and RPC VD in the eyes of 40 patients six months post-discharge for COVID-19 pneumonia. The SCP VD was reduced in the COVID-19 group relative to the control group, just in the whole image. DCP VD showed a decrease in all macular sectors in the COVID-19 cohort relative to the healthy control group. A notable decrease in the RPC VD in the whole image was observed in the COVID-19 cohort. The FAZ area exhibited no substantial difference between the two groups [18]. Garcia et al. reimaged the same patients imaged by Zapata et al. eight months after the first visit. They found lower central retinal VD persistence in patients with moderate and severe COVID-19 pneumonia [28]. Also, Turker et al. reimaged the cohort they had imaged earlier in their first study [19] and compared the results. They indicated that COVID-19 patients exhibited lower VD values compared to the control group in all parafoveal quadrants of both the DCP and SCP during the initial visit, as well as in all parafoveal quadrants of the SCP and the superior and inferior parafoveal quadrants of the DCP at the six-month follow-up. CC flow area values were significantly lower at the 6-month visit than at the initial examination [29]. Compared to healthy subjects, Bajka et al. investigated retinal changes in 168 young adults with a previous COVID-19 infection at least 180 days previously using OCT and OCTA. Only optic disc measurements revealed a higher capillary vessel density in the SARS-CoV-2 group than in healthy subjects [30].

Only a few studies investigated COVID-19-related retinal pathologies with a one-year follow-up. Noor et al.’s study compared 40 post-COVID-19 patients and 40 healthy subjects. OCTA was evaluated at an average of 15.2 +/−6.9 months post-COVID-19 infection. They revealed no significant difference between the two groups in the OCTA parameters [31]. Jevnikar et al. examined the retinal vascular structures of 30 COVID-19 patients at the end of a one-year follow-up using OCTA and compared them with a healthy control group. They found no significant differences between the two groups regarding the deep and superficial capillary plexus density [32].

Some studies have also evaluated peripapillary RNFL thickness and CMT in recovered COVID-19 patients, but the results are controversial. Cennamo et al. reported decreased average RNFL in the COVID-19 group compared to those in the healthy group [16]. Also, Oren et al. found that RNFL thickness in COVID-19 patients was lower but not significantly different from the control group [33]. On the contrary, Burgos-Blasco et al. reported increased peripapillary RNFL thickness in the COVID-19 patients compared with healthy subjects [34]. Beni et al. found that the patients with moderate-intensity SARS-CoV-2 pneumonia had increased peripapillary RNFL thickness [22]. Despite being immune-privileged, the CNS can react to viruses quickly and strongly [35]. Neurotropic viral infections can inflame the brain parenchyma, as evidenced by instances of encephalitis brought on by SARS-CoV-2 [36]. Also, a case report described a 44-year-old Hispanic male patient who developed acute bilateral optic neuritis. Multimodal ocular, orbital, and CNS imaging and paraclinical assessments were carried out to rule out the possibility of demyelinating or another autoimmune disease. These evaluations, as well as other laboratory tests, were negative. Therefore, it has been concluded that his immune system’s response to the COVID-19 viral infection caused the presentation of ONH findings [37]. Our results note an increase in peripapillary RNFL thickness compared to the group without COVID-19, which could suggest an effect of the SARS-CoV-2 virus on the optic nerve. Oren et al. evaluated CMT thicknesses 14-30 days after COVID-19 symptom onset. The mean CMT was significantly higher in the COVID-19 group than in the control group [29]. In contrast, in our study, CMT was lower in the COVID-19 group compared to the control group at all visits. Foveal VD was also lower in both SCP and DCP levels at all control visits compared to the control group. We suggested that decreased VD in the foveal area might result in ischemia and a decreased CMT value. Increased FAZ area results in our study supported this finding.

Our study compared both short- and long-term OCTA findings between healthy subjects and relatively young COVID-19 patients hospitalized with positive PCRs but without a history of hospitalization in the intensive care unit or intubation. All patients had mild to moderate disease. We found that the mean values of FAZ and FAZ PERIM were higher in COVID-19 patients and stayed significantly higher compared to the control group after one year. Another persistent change was decreased foveal and parafoveal nasal SCP VDs in COVID-19 patients during the 1-year follow-up. Also, foveal DCP VD decreased during early visits and persisted during the 1-year follow-up. Even though it was insignificant, there was a progressive decrease in the DCP VD in the perifoveal superior quadrant during the first visits, and it reached significant values at the end of 1 year. These findings could be explained by the multiple pathogenic mechanisms linked to the SARS-CoV-2 infection. The retina has a rich vascular structure, but in contrast to other tissues, the retinal plexi is composed of end arteries without any anastomotic connections. The border that defines the FAZ is comprised of terminal vessels, making it a region especially prone to ischemia alterations [38]. Two main factors may contribute to explaining the decrease in VD. The first possible factor is a decreased blood flow velocity in the retinal vascular bed. Retinal flow impairment may occur locally due to an obstruction within the vessel lumen, mainly by thrombotic occurrences. Enlargement of the diameters of retinal veins and arteries was noted in COVID-19 patients [35]. This finding may be explained by the theory that elevated inflammatory cytokines during the infection contribute to endothelial damage and vessel dilation. Additionally, dilated vascular regions characterized by low blood flow velocity are prone to thrombus formation [36]. Furthermore, vascular dilation may result in diminished vascular flow, leading to a reduced blood cell signal recorded by OCTA, which is later interpreted as decreased OCTA parameters. Second, capillary dropout could result from increased pyroptosis and apoptosis of the endothelial cells in the context of proinflammatory endothelium dysfunction.

We found no differences in the CC parameters of the COVID-19 and the healthy control groups. In contrast to the retina, the CC is characterized by a densely interconnected network of capillaries that exhibit a high degree of anastomosis [39]. This may explain why no significant differences were found in the CC.

For optic disc evaluation, we found that in the COVID-19 group, there was a significant increase in the average, superior, and inferior quadrant RNFL thickness in the first two visits. Still, this increase did not persist at the 12-month visit in the COVID-19 group and became similar to the control group’s. Inflammation caused by the virus may account for the thickening of RNFL in the early phases of the disease. On the contrary, RPC in the temporal quadrant was higher compared to the control group at every visit and stayed higher at the 12-month visit. Unlike the decreased values in macular VD, RPC-VD temporal values were significantly higher in the post-COVID-19 group than in the healthy control group. Ganglion cells and photoreceptors located in the macular area require high oxygen levels. An increase in RPC-VD may be an attempt to compensate for the COVID-19-induced ischemia in the macular area.

As mentioned above, several studies have evaluated microvascular changes in COVID-19 patients. However, retinal involvement has been described inconsistently, and some study findings contradict each other and our results. The vast variation in the time post-COVID-19, the different demographic and concomitant conditions of the patients, and the different disease severity in different studies may explain the inconsistencies in the frequency of abnormal findings among studies.

OCTA results can be affected by multiple factors. The first one is the presence of pre-existing systemic diseases that can affect retinal microvasculature. In our study, analyzed patients were young and without any systemic diseases. The second is supportive oxygen therapy. Twenty-two of our patients had supportive oxygen therapy. Previous studies have demonstrated that retinal VD increases in response to hypoxemia and decreases inversely in the presence of hyperoxemia [40,41]. Oxygen therapy in the acute phase of COVID-19 pneumonia has been considered a possible mechanism. However, direct effects of hyper/hypoxia seem to last just a few minutes [41,42] or even a few days [40]. Our patients’ participation began one month after hospital discharge and oxygen therapy. The persistent reduction observed in the current study, even after 12 months, diminishes the effect of hyperoxemia proposed in previous reports. Our findings indicate that inflammatory and thrombotic events transpire in the retinas of COVID-19 patients. Furthermore, OCTA imaging results in COVID-19 patients corroborate the concept that the pathogenesis of COVID-19 disease includes a microvascular component.

Our study has some limitations. The first of these is the small sample size. Second, no fluorescein angiography was performed to investigate the integrity of the retinal vasculature. However, as previously stated, this study aimed to investigate the changes in retinal microvasculature using the OCTA technique, which could also show retinochoroidal microvascular changes without using contrast material and could be completed in a shorter hospital time during the pandemic period. Third, there were no data for changes in the retinal microvasculature during the acute phase of COVID-19 infection. Fourth, this study focused on relatively young patients with mild to moderate disease severity and without systemic comorbidities; thus, the findings may not reflect the changes observed in patients with severe disease or those with systemic comorbidities. Despite these limitations, the long follow-up period of one year is the strength of our study. In our study, longitudinal testing with repeat imaging at different visits during the course of a year provided information regarding both the short- and long-term effects of COVID-19 on the retinal vasculature.

## 5. Conclusions

Since the end of the pandemic, it has become more important to investigate the long-term effects of COVID-19, and our study demonstrated long-lasting retinal vascular alterations even after 1 year in recovered COVID-19 patients. We demonstrated a sustained reduction in VDs within the central area of the deep and superficial plexuses, alongside increases in the area and perimeter of the FAZ, primarily indicating ischemia. Should persistent microvascular alterations be observed in the retina after 12 months, it can be inferred that other organs and their vascular networks may have long-term damage. Consequently, we believe conducting close follow-ups of post-COVID-19 patients is essential to identify potential long-term ophthalmological and systemic disease sequelae. Long-term multicentered studies with large numbers of patients are needed to clarify long-term choroidal and vascular changes caused by COVID-19.

## Figures and Tables

**Table 1:** Mean age and sex distribution for both groups.

		Control Group (n = 40)	COVID-19 Group (n = 40)	*p*
Sex n (%)	Female	16 (40%)	16 (40%)	1.000
	Male	24 (60%)	24 (60%)	
Age (y) mean ± SD (Min-Max)		40.1 ± 11.2 (21–58)	42.9 ± 10.9 (18–58)	0.053

**Table 2:** Comparison of RNFL and peripapillary vessel density parameters.

		Control Group (n = 40)	COVID-19 Group (n = 40)	
		Mean ± SD	Mean ± SD	*p*
RNFL thickness—Average (µm)	1st month	113.9 ± 10.7	118.9 ± 11.9	0.014 *
	4th month	114.1 ± 12.2	118.9 ± 12.3	0.020
	12th month	114.8 ± 11.2	116.3 ± 12.1	0.213 *
	p^a^	0.45	0.01	
RNFL thickness—Superior (µm)	1st month	133.1 ± 13.8	141.4 ± 13.4	0.001 ^#^
	4th month	133.8 ± 13.9	141.0 ± 14.3	0.003 ^#^
	12th month	133.6 ± 14.1	136.1 ± 14.0	0.057 ^#^
	p^a^	0.79	0.008	
RNFL thickness—inferior (µm)	1st month	143.0 ± 15.8	150.8 ± 21.2	0.021 *
	4th month	143.5 ± 16.3	150.3 ± 20.9	0.024 *
	12th month	144.1 ± 16.8	147.3 ± 24.2	0.074 *
	p^a^	0.86	0.012	
RNFL thickness—nasal (µm)	1st month	103.7 ± 14.4	108.2 ± 17.2	0.122
	4th month	104.5 ± 14.8	109.2 ± 17.7	0.433 *
	12th month	104.1 ± 15.0	106.0 ± 17.9	0.613 *
	p^a^	0.75	0.52	
RNFL thickness—temporal (µm)	1st month	76.1 ± 9.5	77.9 ± 9.5	0.307 ^#^
	4th month	76.5 ± 9.4	77.2 ± 9.6	0.543 ^#^
	12th month	76.4 ± 9.5	76.9 ± 9.4	0.642 ^#^
	p^a^	0.83	0.69	
RPC SV VD (%)—whole image-	1st month	49.5 ± 2.2	50.2 ± 2.2	0.121 ^#^
	4th month	49.8 ± 2.3	50.2 ± 2.0	0.075 ^#^
	12th month	50.0 ± 2.4	50.2 ± 2.6	0.118 ^#^
	p^a^	0.67	0.88	
RPC SV VD (%)—inside disc	1st month	51.6 ± 3.4	51.1 ± 3.1	0.356 ^#^
	4th month	51.5 ± 3.5	51.3 ± 3.5	0.595 ^#^
	12th month	51.1 ± 3.6	50.5 ± 3.4	0.078 ^#^
	p^a^	0.87	0.63	
RPC SV VD (%)—peripapillary	1st month	51.5 ± 2.3	52.2 ± 2.6	0.147 ^#^
	4th month	51.9 ± 2.5	52.3 ± 2.7	0.097 ^#^
	12th month	51.8 ± 2.6	52.2 ± 3.1	0.195 ^#^
	p^a^	0.84	0.81	
RPC SV VD (%)—Superior	1st month	51.7 ± 3.3	52.6 ± 3.9	0.161 ^#^
	4th month	51.9 ± 3.4	52.8 ± 3.6	0.121 *
	12th month	52.0 ± 3.5	52.6 ± 3.8	0.179 *
	p^a^	0.71	0.92	
RPC SV VD (%)—Inferior	1st month	52.5 ± 3.7	52.7 ± 3.8	0.846 *
	4th month	52.7 ± 4.0	53.0 ± 4.2	0.471 *
	12th month	52.6 ± 4.2	52.4 ± 5.5	0.867 *
	p^a^	0.76	0.79	
RPC SV VD (%)—Nasal	1st month	49.6 ± 2.9	49.2 ± 3.5	0.390 *
	4th month	49.5 ± 3.1	49.4 ± 3.8	0.687 *
	12th month	49.5 ± 3.3	49.4 ± 3.9	0.948 *
	p^a^	0.91	0.89	
RPC SV VD (%)—Temporal	1st month	53.7 ± 4.4	54.9 ± 2.8	0.003 *
	4th month	53.3 ± 3.1	54.8 ± 2.8	0.003 *
	12th month	53.6 ± 3.9	55.7 ± 3.9	<0.001 *
	p^a^	0.88	0.62	

^#^ Student *t*-Test, * Mann–Whitney U Test, p^a^ Comparisons were performed using repeated measures analysis of variance with Bonferroni adjustment; statistically significant when *p* < 0.016. In pairwise comparisons for COVID-19 group, RNFL thickness average p values for 1st month–4th month: 0.89, 4th month–12th month: 0.015, 1st month–12th month: 0.014; RNFL thickness superior 1st–4th month p: 0.92, 4th–12th month: 0.01, 1st–12th month: 0.009; RNFL thickness inferior *p* values for 1st–4th month: 0.77, 4th–12th month: 0.009, 1st–12th month: 0.011. RNFL: retinal nerve fiber layer, RPC: radial peripapillary capillary, SV: small vessel, VD: vessel density, SD: standard deviation.

**Table 3:** Comparison of FAZ and microvascular flow parameters and CMT.

		Control Group (n = 40)	COVID-19 Group (n = 40)	
		Mean ± SD	Mean ± SD	*p*
Foveal avascular zone (FAZ) parameters and central macular thickness (CMT)				
FAZ area (mm^2^)	1st month	0.24 ± 0.10	0.29 ± 0.09	0.002 ^#^
	4th month	0.25 ± 0.10	0.32 ± 0.35	0.002 *
	12th month	0.24 ± 0.21	0.30 ±0.11	0.002 *
	p^a^	0.85	0.78	
FAZ perimeter (mm)	1st month	1.84 ± 0.44	2.07 ± 0.36	0.002 ^#^
	4th month	1.85 ± 0.41	2.08 ± 0.37	0.003 ^#^
	12th month	1.86 ± 0.42	2.07 ± 0.40	0.005 ^#^
	p^a^	0.75	0.89	
CMT (μm)	1st month	256.2 ± 22.1	241.4 ± 20.9	<0.001 ^#^
	4th month	258.5 ± 21.0	242.5 ± 20.4	0.001 ^#^
	12th month	255.0 ± 21.5	243.0 ± 21.6	0.001 ^#^
	p^a^	0.88	0.69	
Microvascular flow parameters				
Outer retinal flow area (mm^2^)	1st month	0.48 ± 0.30	0.58 ± 0.42	0.315 *
	4th month	0.47 ± 0.33	0.49 ± 0.37	0.675 *
	12th month	0.49 ± 0.35	0.50 ± 0.39	0.619 *
	p^a^	0.47	0.16	
Choriocapillaris flow area (mm^2^)	1st month	2.13 ± 0.11	2.11 ± 0.11	0.397 ^#^
	4th month	2.12 ± 0.12	2.13 ± 0.11	0.878 ^#^
	12th month	2.12 ± 0.12	2.10 ± 0.11	0.119 ^#^
	p^a^	0.77	0.54	

^#^ Student *t*-Test, * Mann–Whitney U Test, p^a^ Comparisons were performed using repeated measures analysis of variance with Bonferroni adjustment; statistically significant when *p* < 0.016. FAZ: foveal avascular zone, CMT: central macular thickness, SD: standard deviation.

**Table 4:** Comparison of macular SCP VD parameters.

		Control Group (n = 40)	COVID-19 Group (n = 40)	
Vessel Density; SCP Flow (%)		Mean ± SD	Mean ± SD	*p*
Whole image	1st month	51.4 ± 2.1	50.6 ± 2.9	0.085
	4th month	51.2 ± 2.3	50.8 ± 2.8	0.464 *
	12th month	51.1 ± 2.4	50.7 ± 3.2	0.233 ^#^
	p^a^	0.69	0.77	
Fovea	1st month	24.9 ± 7.5	20.0 ± 5.7	<0.001 ^#^
	4th month	24.5 ± 6.8	19.8 ± 5.9	<0.001 ^#^
	12th month	24.2 ± 7.0	20.0 ± 6.1	<0.001 ^#^
	p^a^	0.62	0.83	
Parafoveal whole density	1st month	53.8 ± 2.3	52.8 ± 3.8	0.071
	4th month	53.5 ± 2.8	53.4 ± 3.0	0.775 *
	12th month	53.3 ± 2.9	53.2 ± 3.3	0.273 ^#^
	p^a^	0.81	0.56	
* Temporal	1st month	53.3 ± 2.2	52.4 ± 3.8	0.097 ^#^
	4th month	53.2 ± 3.1	52.9 ± 3.3	0.959 *
	12th month	53.0 ± 3.2	53.1 ± 3.7	0.878 *
	p^a^	0.86	0.74	
* Superior	1st month	54.4 ± 2.7	53.7 ± 3.9	0.252 ^#^
	4th month	54.2 ± 2.9	54.3 ± 3.2	0.791 ^#^
	12th month	54.0 ± 3.1	54.1 ± 3.3	0.517 ^#^
	p^a^	0.77	0.62	
* Nasal	1st month	53.4 ± 2.9	51.7 ± 3.7	0.009 ^#^
	4th month	52.9 ± 3.0	52.3 ± 2.8	0.047 ^#^
	12th month	52.7 ± 3.1	52.2 ± 3.2	0.042 ^#^
	p^a^	0.82	0.45	
* Inferior	1st month	54.2 ± 2.5	53.3 ± 4.9	0.895 *
	4th month	54.0 ± 3.4	54.1 ± 4.2	0.879 ^#^
	12th month	53.8 ± 3.5	53.6 ± 4.3	0.726 *
	p^a^	0.63	0.66	
Perifoveal whole density	1st month	52.1 ± 2.2	51.2 ± 2.9	0.067 ^#^
	4th month	51.9 ± 2.7	51.5 ± 3.0	0.477 *
	12th month	51.8 ± 2.8	51.5 ± 3.4	0.308 ^#^
	p^a^	0.61	0.89	
* Temporal	1st month	48.7 ± 2.5	47.6 ± 3.3	0.078 ^#^
	4th month	48.5 ± 2.9	47.5 ± 3.1	0.041 ^#^
	12th month	48.4 ± 3.1	47.7 ± 3.6	0.342 *
	p^a^	0.58	0.35	
* Superior	1st month	51.7 ± 2.8	51.0 ± 3.3	0.237 ^#^
	4th month	51.5 ± 3.2	51.2 ± 3.4	0.410 ^#^
	12th month	51.4 ± 3.4	51.1 ± 3.9	0.587 ^#^
	p^a^	0.85	0.86	
* Nasal	1st month	55.5 ± 2.0	54.8 ± 2.5	0.125 ^#^
	4th month	55.3 ± 2.4	55.3 ± 2.7	0.735 ^#^
	12th month	55.2 ± 2.6	55.5 ± 2.9	0.661 ^#^
	p^a^	0.76	0.88	
* Inferior	1st month	52.0 ± 3.4	51.5 ± 3.5	0.453 *
	4th month	51.9 ± 3.7	51.9 ± 3.8	0.921 *
	12th month	51.8 ± 4.0	51.7 ± 4.3	0.874 *
	p^a^	0.81	0.78	

^#^ Student *t*-Test, * Mann–Whitney U Test, p^a^ Comparisons were performed using repeated measures analysis of variance with Bonferroni adjustment; statistically significant when *p* < 0.016. VD: vessel density, SCP: superficial capillary plexus, SD: standard deviation.

**Table 5:** Comparison of macular DCP VD parameters.

		Control Group (n = 40)	COVID-19 Group (n = 40)	
Vessel Density; DCP Flow (%)		Mean ± SD	Mean ± SD	*p*
Whole image	1st month	54.1 ± 5.5	53.6 ± 4.7	0.544 ^#^
	4th month	54.0 ± 5.0	53.5 ± 4.3	0.458 ^#^
	12th month	53.7 ± 5.4	52.4 ± 5.6	0.083 ^#^
	p^a^	0.34	0.42	
Fovea	1st month	41.6 ± 7.6	37.7 ± 7.4	0.005 ^#^
	4th month	41.2 ± 7.2	36.9 ± 6.9	0.001 ^#^
	12th month	40.8 ± 7.4	36.6 ± 7.8	0.001 ^#^
	p^a^	0.29	0.58	
Parafoveal whole density	1st month	56.4 ± 4.2	56.3 ± 3.4	0.832 *
	4th month	56.2 ± 3.8	56.1 ± 3.3	0.525 *
	12th month	55.7 ± 4.0	55.1 ± 4.1	0.105 ^#^
	p^a^	0.85	0.72	
* Temporal	1st month	57.2 ± 4.3	57.0 ± 3.8	0.541 *
	4th month	57.0 ± 3.6	57.1 ± 3.2	0.808 ^#^
	12th month	56.8± 3.9	56.3 ± 4.0	0.162 ^#^
	p^a^	0.56	0.29	
* Superior	1st month	55.8 ± 4.5	55.9 ± 3.9	0.923 *
	4th month	55.4 ± 4.1	55.6 ± 3.9	0.706 ^#^
	12th month	55.3 ± 4.4	54.5 ± 4.2	0.124 *
	p^a^	0.73	0.31	
* Nasal	1st month	56.8 ± 4.2	56.9 ± 3.4	0.887 *
	4th month	56.5 ± 3.6	56.7 ± 3.2	0.649 *
	12th month	55.9 ± 4.0	55.8 ± 4.1	0.156 *
	p^a^	0.62	0.33	
* Inferior	1st month	55.5 ± 4.7	55.3 ± 3.8	0.887 *
	4th month	55.3 ± 4.0	55.1 ± 3.9	0.617 ^#^
	12th month	55.1 ± 4.5	53.8 ± 4.6	0.090 ^#^
	p^a^	0.53	0.12	
Perifoveal whole density	1st month	55.7 ± 5.7	55.2 ± 5.1	0.522 *
	4th month	55.5 ± 5.0	55.2 ± 4.6	0.567 ^#^
	12th month	55.1 ± 5.5	54.0 ± 6.0	0.107 ^#^
	p^a^	0.74	0.17	
* Temporal	1st month	57.3 ± 4.5	57.4 ± 4.3	0.964 *
	4th month	57.0 ± 4.0	56.7 ± 4.0	0.407 ^#^
	12th month	56.9 ± 4.6	55.9 ± 5.2	0.176 *
	p^a^	0.65	0.25	
* Superior	1st month	55.3 ± 6.4	54.1 ± 5.5	0.275 ^#^
	4th month	54.79± 5.5	54.4 ± 5.3	0.384 ^#^
	12th month	55.1 ± 6.1	52.4 ± 6.6	0.014 ^#^
	p^a^	0.58	0.023	
* Nasal	1st month	54.3 ± 6.3	53.5 ± 5.5	0.450 ^#^
	4th month	54.1 ± 5.5	54.0 ± 5.3	0.785 ^#^
	12th month	54.2 ± 6.2	53.0 ± 6.5	0.310 ^#^
	p^a^	0.58	0.65	
* Inferior	1st month	56.0 ± 6.5	55.7 ± 6.6	0.749 *
	4th month	55.9 ± 5.9	55.7 ± 5.8	0.741 *
	12th month	55.8 ± 6.1	54.4 ± 7.2	0.216 *
	p^a^	0.66	0.47	

^#^ Student *t*-Test, * Mann–Whitney U Test, p^a^ Comparisons were performed using repeated measures analysis of variance with Bonferroni adjustment; statistically significant when *p* < 0.016. VD: vessel density, DCP: deep capillary plexus, SD: standard deviation.

## Data Availability

The article encompasses the original contributions outlined in the study. Additional enquiries may be sent to the corresponding author.
